# Diagnostic accuracy of using multiple cytokines to predict aldosterone-producing adenoma

**DOI:** 10.1038/s41598-023-32558-9

**Published:** 2023-04-07

**Authors:** Fei Qin, Hong Wen, Xiaoge Zhong, Yajin Pan, Xiaomei Lai, Tingting Yang, Jing Huang, Jie Yu, Jianling Li

**Affiliations:** 1 Department of Cardiology, The First Affiliated Hospital of Guangxi Medical University, Nanning, 530021 Guangxi China; 2 Mobile Post-Doctoral Stations of Guangxi Medical University, Nanning, 530021 Guangxi China

**Keywords:** Immunology, Medical research, Pathogenesis

## Abstract

Here**,** we aimed to study the important cytokines in plasma to identify the aldosterone-producing adenoma (APA). 19 unilateral primary aldosteronism (UPA) patients and 19 healthy people were divided into UPA group and Control group, and the serum of bilateral adrenal veins and inferior vena cava collected by adrenal blood sampling (AVS) in UPA patients and the serum from the healthy subjects were all used to detect multiple cytokines by Luminex immunoassays. Additionally, The UPA patients subjected to laparoscopic adrenalectomy were divided into different groups by pathological results for further study. According our results, IP-10, CXCL9 and RANTES were significantly higher in UPA group compared with control group, and the combination of the three cytokines have significant predictive power for predicting UPA, while the correlational analyses demonstrated that IP-10 and CXCL9 were positively correlated with BP and HR, while EGF was positively correlated with HDL. Additionally, IL-1b was suggested to be the most potential diagnostic biomarker to discriminate the APA and unilateral adrenal hyperplasia (UAH). The present findings might suggest a possibility of IP-10, CXCL9 and RANTES served as a sign to help UPA diagnosis and finally used to assist the diagnosis of APA, while IL-1b was suggested to be the most potential diagnostic biomarker to identify the APA from the UAH patients.

## Introduction

Aldosterone-producing adenoma (APA), as the main type of primary aldosteronism (PA), accounts for about 60–85% of PA^1^, which has serious consequences for the increase of blood pressure and the development of cardiovascular and renal diseases^2^, but the pathogenesis of APA was not clear yet. APA is characterized by high secretion of aldosterone (ALD), the secretion of which is considered as a modulator of immunity. Studies have shown that aldosterone can not only promote the secretion of inflammatory factors, like transforming growth factor-β (TGF-β), CTGF, CCL2, Smad2/3, and nuclear factor-kappaB (NF-kB)^3–5^, but also induce inflammatory responses in various organs and tissues, including kidney, cardiovascular system and artery vessels^6^. Furthermore, the existence of plentiful amounts of immune cells and active immune effects in the adrenal gland have been demonstrated by single-cell RNA sequencing (scRNA-seq)^7^, which implicated the pathogenic mechanism of adrenal tumors might be regulated by immune cells.

Cytokines are secreted by multiple cell types, including immune and non-immune cells, like fibroblasts, endothelium cells, epithelium cells, macrophages, lymphocytes, cancer cells, etc.^8^, which possess multiple biological function, not only modulating immune response, inflammation process, but also regulating cell proliferation and differentiation^9^. Some researches have demonstrated the role of cytokines and their receptors in regulating tumor microenvironment (TME) and different solid tumors formation^10–12^. Some researchers have found that5-hydroxytryptamine (5-HT) secreted by mast cells could promote the mRNA expression of aldosterone synthase (CYP11B2) and the secretion of aldosterone^13^, and interleukin-1b (IL-1b) secreted by macrophages has been confirmed to stimulate adrenal cortical cell proliferation^14,15^, but only a few previous studies have focused on the correlation of inflammation cytokine levels with APAs, and data regarding cytokine profiling in the APA are merely limited to a small subset of cytokines, which leads to a lack of knowledge about the changes of plasma cytokines in patients with APA and no effective inflammatory factors to detect the presence of this disease.

The HISTALDO consensus has made new classification in unilateral primary aldosteronism (UPA), and categorised UPA into classic and non-classic histopathological forms^16^, among which the main types of unilateral primary aldosteronism were aldosterone-producing adenoma (APA) as classic histopathological type and unilateral adrenal hyperplasia (UAH) as non-classic histopathological type^1^, which are usually cured by unilateral adrenalectomy. Although CT imaging combined with AVS was generally used to establish primary aldosteronism subtype, the accuracy of which was reached 90% in differentiating unilateral primary aldosteronism from bilateral primary aldosteronism^2,17,18^. However, CT scan with AVS operation could not distinguish the unilateral primary aldosteronism subtypes, and the final diagnosis depend on histopathologic result, and the pathology types of APA and UAH were adrenocortical adenoma and adrenocortical hyperplasia respectively^19,20^. As we all know, pathological diagnosis usually takes a long time, and in order to further improve the diagnostic efficiency of APA, we wanted to find some inflammatory factors to predict APA more accurately.

Therefore, in order to study the relationship between the occurrence and development of APA and the immune system as well as cytokines, and to find the potential plasma biomarkers to predict APA, we will conduct the detection of cytokines based on the MILLIPLEX immune kit and Luminex detection instrument, and detect 48 cytokines in serum samples from unilateral PA patients.

## Materials and methods

### Study population

19 consecutive patients diagnosed with unilateral PA served as UPA group and 19 healthy people with matching sex, age were enrolled served as control group were collected from The First Affiliated Hospital of Guangxi Medical University between November 2019 and December 2020. Adrenal blood sampling (AVS) was performed to identity the laterality of aldosterone hypersecretion to guide surgical operation, the lateralization index (LI) > 2 without adrenocorticotropic hormone stimulation was served as the standard cutoff to identify the laterality. The LI is defined as the aldosterone to cortisol concentration ratio of the dominant side from one side of the adrenal vein divided by that of the non-dominant side from another side of the adrenal vein, and aldosterone level in the dominant side is significantly elevating than that in non-dominant side^21,22^. Therefore, the patients of UPA group were all subject to AVS to clarify the laterality of aldosterone hypersecretion and surgical site, meantime the plasma of adrenal veins and inferior vena cava was collected to further detect the inflammation cytokines inside. The serum collected from adrenal veins and inferior vena cava was divided into three subgroups: the Middle group (the serum from inferior vena cava), the Same group (the serum from the dominant adrenal vein) and Opposite group (the serum from the non-dominant adrenal vein) according to the AVS result. The dominant side were identified when one side possesses cortisol-normalized ALD levels over 2 times than the other side and meantime the adrenal occupying lesion showed the same side of adrenal gland with the AVS result by adrenal enhancement CT, or identified as non-dominant side. Furthermore, 18 patients in UPA group subjected to laparoscopic adrenalectomy were divided into Unilateral adrenal adenoma (APA) (n = 13) and Unilateral adrenal hyperplasia (UAH) (n = 5) according to the pathological materials. Inclusion criteria for UPA group: (1) The suspected PA patients conducted the measurement of plasma renin activity (PRA) and plasma aldosterone concentration (PAC) to calculate ARR value (PAC to PRA ratio) for primary screening, and ARR ≥ 30 was chosen as entry point for further confirmatory test. (2) Confirmatory test: PAC > 100 pg/ml after saline loading or aldosterone levels decreasing by less than 30% after 50 mg captopril administration were considered as positive result for PA diagnosis. (3) Enhanced CT scanning showed unilateral adrenal occupying lesion. (4) The lateralization of aldosterone secretion decided by AVS was in accordance with the CT occupying side. Exclusion criteria: (1) Patients with severe liver, kidney, heart dysfunction, hyperthyroidism and malignant tumor (2) Patients with trauma, and mental diseases that can’t cooperate with operators, and pregnant women and lactating women (3) Patients with the use of steroids and immunosuppressors. This study was performed in line with the principles of the Declaration of Helsinki. Approval was granted by the Ethics Committee of Guangxi Medical University and the informed consent was obtained from all individual participants.

### Clinical materials

General clinical materials such as gender, age, height, weight, blood pressure (BP), heart rate (HR), etc., as well as biochemical indicators such as aldosterone levels, plasma potassium value, and renal function, and other important clinical indicators were collected, and BMI was calculated using height and weight indicators. Moreover, the collection of pathological data was also conducted for further grouping and comparison.

### Sample collection

Before the AVS, β receptor blockers, angiotensin-converting enzyme inhibitor (ACEI), and angiotensin receptor blocker (ARB) were weaned over for at least 2 weeks, diuretics for at least 4 weeks, and spironolactone for at least 6 weeks, and was no use of ACTH stimulation during or before the AVS operation. 5 ml blood samples from left adrenal vein, right adrenal vein and inferior vena cava were obtained through catheters in APA group under local anesthesia, while the blood of control group was drawn from elbow veins. Then, all the blood samples were centrifuged at 3000 g speed for 10 min, and supernatant was collected and stored in a refrigerator at -80℃ for further testing.

### Cytokine measurements

Human Cytokine Antibody-immobilized Magnetic Bead Panel (HCYTA-60 K-PX48, Millipore, MA, USA) was used to detect the concentration of 48 cytokines including sCD40L, EGF, Eotaxin, FGF-2, FLT-3L, Fractalkine, G-CSF, GM-CSF, GROa, IFNa2, IFNy, IL-1a, IL-1b, IL-1RA, IL-2, IL-3, IL-4, IL-5, IL-6, IL-7, IL-8, IL-9, IL-10, IL-12p40, IL-12p70, IL-13, IL-15, IL-17A, IL-17E, IL-17F, IL-18, IL-22, IL-27, IP-10, MCP-1, MCP-3, M-CSF, MDC, CXCL9, MIP-1a, MIP-1b, PDGF-AA, PDGF-AB-BB, RANTES, TGFa, TNFa, TNFb, VEGF-A according to the standard detection protocol, and MILLIPLEX Analyzer.V5.1 software was used for cytokines concentration analysis.

### Histopathology

Successive paraffin-embedded adrenal tissue sections with 3-µm thick were cut to conducted histopathological investigation of the change of adrenal RANTES, CXCL9, and IP10 protein between the UPA and normal adrenal gland. The UPA samples divided into two type including adenoma and hyperplasia were immunostained with IL1 antibody to study the expression difference. The sections were deparaffinized, rehydrated, and repaired, then endogenous peroxidase activity was blocked by treatment with 3.0% H2O2 before antibody staining, then the histological sections were incubated with the primary antibody and secondary antibody, respectively, followed by tissue counterstaining with hematoxylin and dehydration, neutral balsam mounting in the end. The adrenal sections were observed in a digital microscope (Olympus, Tokyo, Japan) and were evaluated by calculating the average optical density using Image-Pro Plus 6.

### Statistical analyses

All statistical analyses were performed using SPSS version 16.0 software (SPSS Inc, Chicago, IL, USA). Normally distributed variables were presented as mean and standard deviation (SD), while non-normally distributed variables were presented as median and range. Two-group comparisons were performed with Mann–Whitney test or Student’s t-test according to the data distribution, while three-group comparisons from the subgroups of UPA group were conducted by Friedman test using randomized block design. However, the Mann–Whitney test of independent samples was used for comparison between the comparison of Adenoma group and Hyperplasia group for only 5 cases included in the Hyperplasia group and normality test could not be performed. Additionally, Pearson’s correlation coefficient was used for correlation analysis. P < 0.05 were considered statistically significant. Based on R language (version 4.0.3), receiver operating characteristic (ROC) curves were calculated for evaluation of predictive ability of the important cytokines.

## Results

### Baselines comparison

The basic information of all the subjects was showed in Table 1. By comparing the baseline clinical characteristics of the UPA group and the control group, we found that the levels of SBP, DBP, BMI, HR and TG in the UPA group were significantly higher than those in the control group, while the levels of HDL were significantly lower than those in the control group (p < 0.05). However, there was no statistical significance in age, LDL, TC and Cr levels between the two groups (p > 0.05).

**Table 1:** Baseline clinical characteristics of the study cohort.

	UPA(n = 19)	Control(n = 19)	P
Gender (male: female)	5:14	5:14	
Age	47.9 ± 11.3	42.7 ± 10.6	0.148
SBP (mmHg)	148.0 (131.0 ~ 164.0)	115.0 (107.0 ~ 123.0)	0.000
DBP (mmHg)	93.0 (84.0 ~ 103.0)	70.0 (66.0 ~ 75.0)	0.000
BMI (kg/m^2^)	24.4 ± 3.7	21.8 ± 2.8	0.019
HR (BPM)	84.8 ± 11.8	74.5 ± 7.0	0.002
TG (mmol/L)	1.4 (0.86 ~ 1.64)	1.0 (0.71 ~ 1.14)	0.02
HDL (mmol/L)	1.2 ± 0.2	1.5 ± 0.3	0.004
LDL (mmol/L)	2.9 (2.2 ~ 3.9)	2.6 (2.1 ~ 2.9)	0.154
TC (mmol/L)	4.7 ± 1.0	5.1 ± 0.6	0.196
FBG (mmol/L)	4.9 (4.5 ~ 5.5)	5.16 (4.88 ~ 5.32)	0.246
Cr (μmoI/L)	65.5 ± 17.5	64.6 ± 14.5	0.865

SBP: systolic blood pressure; DBP: diastolic blood pressure; BMI: body mass index; HR: heart rate; TG: triglyceride; HDL: high density lipoprotein; LDL: low density lipoprotein; TC: total cholesterol; FBG: fasting blood glucose; Cr: creatinine.

P < 0.05 was considered significant.

### Comparison of 48 cytokines between UPA group (inferior vena cava) and control group

By comparing 48 cytokine levels of UPA group and control group (Table2), we obtained 16 cytokines showing statistical significance, among which IP-10, CXCL9 and RANTES were higher in APA group than in control group(p < 0.05), while the other 13 cytokines including IL-1a, IL-1b, IL-2, IL-5, IL-8, MCP-1, MIP-1b, PDGF- AB-BB, TGFa, TNFb, VEGF-A, EGF and IL-17A were lower in UPA group than in control group(p < 0.05). The remaining cytokines were not statistically significant between the two groups (p > 0.05).

**Table 2:** Serum levels of cytokines between UPA and control groups.

	Middle (n = 19)	Control (n = 19)	P
CXCL9 (pg/ml)	1220 (841.2 ~ 1883)	810.9 (528.7 ~ 1494)	0.017
IP-10 (pg/ml)	33.3 (23.7 ~ 50.4)	20.9 (11.5 ~ 24.9)	0.001
RANTES (pg/ml)	3959 (3105 ~ 4639)	2984 (2686 ~ 3502)	0.034
IL-1a (pg/ml)	7.1 (4.5 ~ 9.2)	11.53 (9.18 ~ 26.54)	0.001
IL-1b (pg/ml)	4.7 (3.0 ~ 7.9)	9.7 (3.8 ~ 15.0)	0.043
IL-2 (pg/ml)	0.5 (0.5 ~ 1.1)	1.6 (0.8 ~ 5.3)	0.022
IL-8 (pg/ml)	4.3 (2.3 ~ 7.2)	79.5 (50.0 ~ 122.2)	0.000
IL-5 (pg/ml)	2.7 (1.5 ~ 5.0)	6.8 (4.0 ~ 12.2)	0.006
MCP-1 (pg/ml)	268.5 ± 91.3	350.2 ± 127.4	0.029
MIP-1b (pg/ml)	32.8 (28.3 ~ 37.3)	45.8 (33.0 ~ 50.0)	0.008
PDGF-	15,155.1 ± 3872.4	18,904.8 ± 5158.3	0.016
AB-BB (pg/ml)			
TGF-a (pg/ml)	4.5 (3.4 ~ 11.4)	9.7 (7.3 ~ 13.7)	0.002
TNF-b (pg/ml)	6.0 (2.5 ~ 12.4)	15.4 (6.9 ~ 34.1)	0.005
VEGF-A (pg/ml)	39.0 (23.6 ~ 50.8)	206.5 (147.0 ~ 342.9)	0.000
EGF (pg/ml)	68.8 (33.7 ~ 103.1)	172.9 (91.0 ~ 283.3)	0.001
IL-17A (pg/ml)	2.4 (1.6 ~ 4.1)	3.8 (2.7 ~ 9.2)	0.023

P < 0.05 was considered significant.

### The ability of IP-10, CXCL9, and RANTES in plasma to diagnose the UPA patients

**Figure 1:**
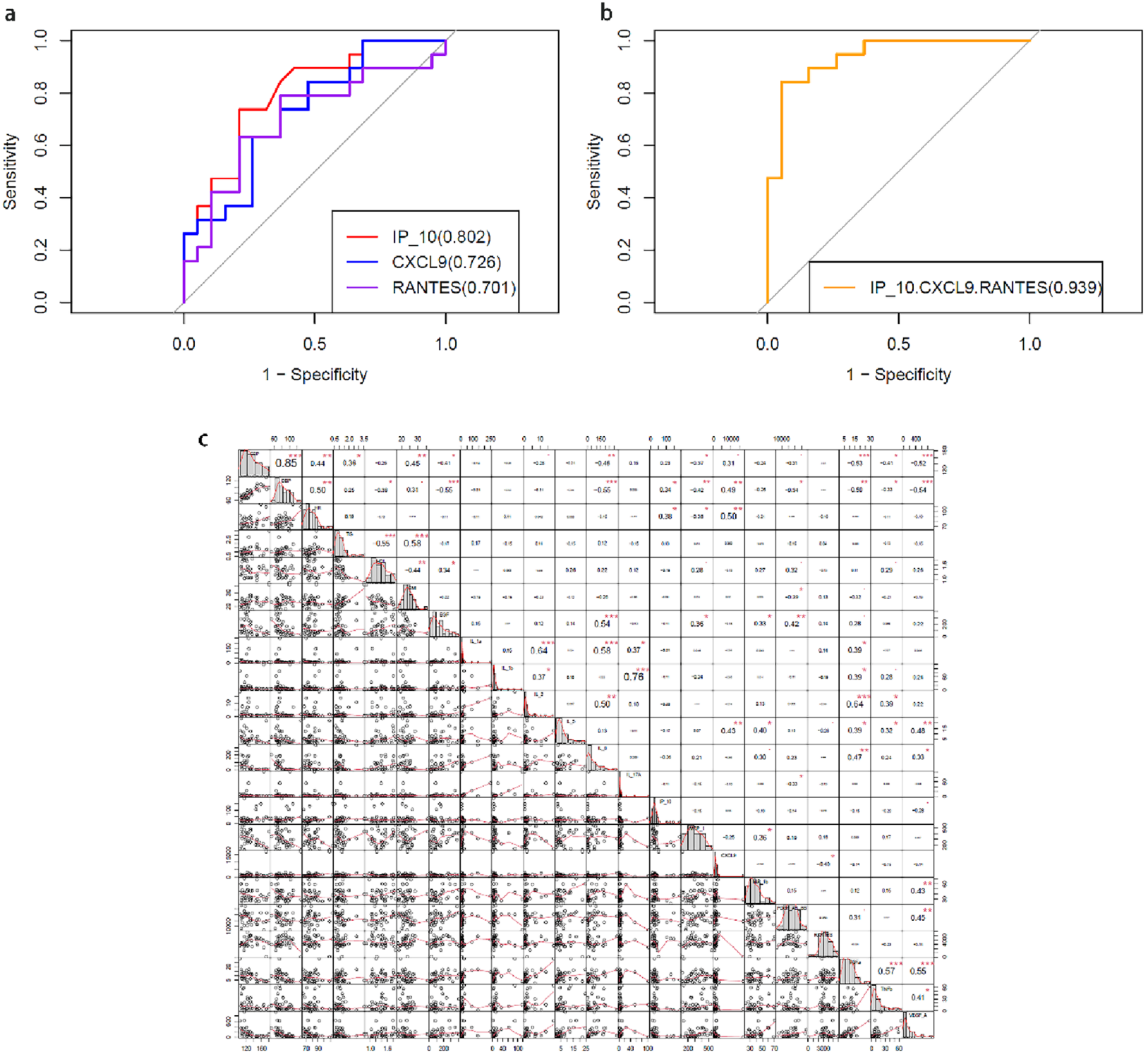
(**a**) ROC curves of single-indicator analysis of IP-10, CXCL9 and RANTES; (**b**) ROC curves of combined analysis of IP-10, CXCL9 and RANTES. (**c**)The correlational analyses in the 16 statistically significant cytokines with the clinical indicators in the comparison of UPA group and control group.

The ability of the IP-10, CXCL9, and RANTES to discriminate UPA patients from healthy control subjects was evaluated using ROC curve analysis (Fig. 1a,b). According to the result, we found that IP-10 owned the highest AUC value of 0.802, while IL-15 had the lowest AUC value of 0.506. In order to improve the probability of prediction, we combined the three cytokines and obtained the maximum AUC value of 0.939, more than 90%, which has high diagnostic value.

### The correlational analyses in clinical indicators and some cytokines

**Figure 2:**
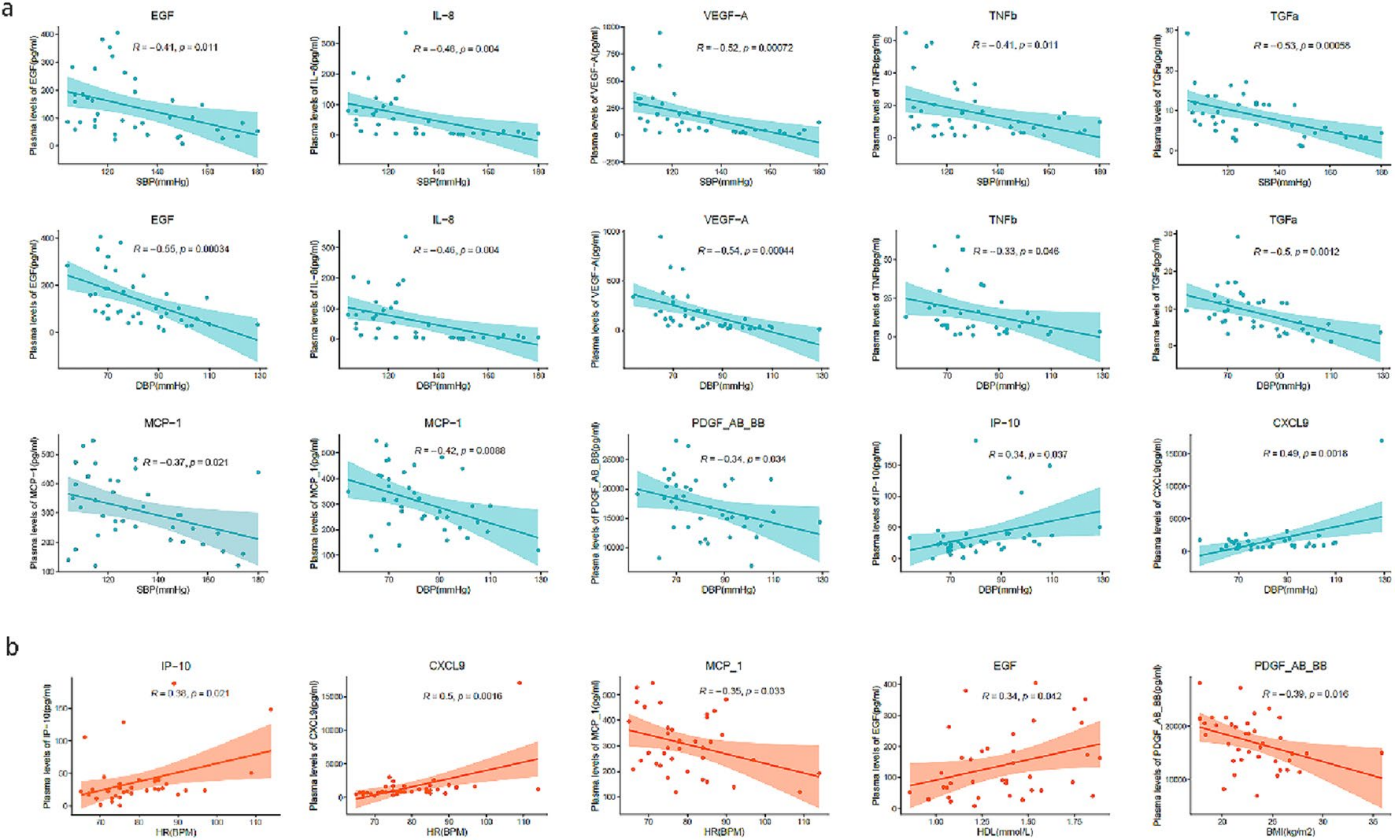
The correlation between the clinical indicators and some cytokines. (**a**) The correlation between the BP value and some significant cytokines. (**b**) The correlation between the HR, HDL, and BMI and the cytokines.

The correlational analyses were conducted between the important cytokines and the clinical indicators (Fig. 1c), and the part important results of correlation analysis were shown in the Fig. 2a,b. The results showed that the cytokines of EGF, IL-8, VEGF-A, TNF-β, TGF-α, MCP-1, and PDGF-AB-BB were negatively correlated with BP. Moreover, IP-10 and CXCL9 were positively correlated with BP and HR, but MCP-1 was negatively correlated with HR. EGF were positively correlated with HDL, but PDGF-AB-BB was were negatively correlated with HDL.

### The comparison of three subgroups of UPA groups in cytokines levels

The pairwise comparison between the three groups were conducted by Friedman test in Table 3. The results showed that FLT-3L, G-CSF, IL-15, the M-CSF, MDC in dominant side (the Same group) and the non-dominant side (the Opposite group) were all higher than the inferior vena cava (the Middle group), while IL-12p40 and MCP-1 levels were higher only in the dominant side than inferior vena cava groups. In addition, the level of Eotaxin cytokine in the non-dominant group was higher than that in the inferior vena cava (P < 0.05). However, the levels of all cytokines were not statistically significant between the dominant and non-dominant groups (P > 0.05).

**Table 3:** Cytokines levels of three subgroups.

	Middle (n = 19)	Opposite (n = 19)	Same (n = 19)
Eotaxin (pg/ml)	72.0 (52.0 ~ 90.2)	62.1 (48.1 ~ 75.3)*	62.4 (50.7 ~ 84.7)
FLT-3L (pg/ml)	13.7 ± 7.6	17.0 ± 7.7*	16.7 ± 8.2*
G-CSF (pg/ml)	15.5 (7.8 ~ 26.5)	22.8 (13.8 ~ 30.1)*	25.2 (13.8 ~ 30.9)*
IL-12p40 (pg/ml)	49.9 (33.1 ~ 65.2)	54.1 (38.7 ~ 70.1)	56.9 (40.1 ~ 66.6)*
IL-15 (pg/ml)	3.6 (3.1 ~ 5.5)	4.6 (4.3 ~ 6.1)*	4.7 (3.4 ~ 6.7)*
MCP-1 (pg/ml)	253.3 (199.8 ~ 317.8)	281.3 (230.0 ~ 341.6)	287.8 (239.0 ~ 385.9)*
M-CSF (pg/ml)	56.0 (17.4 ~ 101.9)	72.4 (35.9 ~ 144.5)*	101.9 (27.8 ~ 190.1)*
MDC (pg/ml)	375.3 ± 96.95	462.894 ± 127.8*	476.5 ± 110.5*

*P < 0.05 are considered significant when compared with the Middle group, because the comparison between the Opposite and Same groups didn’t show any statistically significant in all the cytokines.

### Comparison of baseline data between adenoma and hyperplasia groups

**Figure 3:**
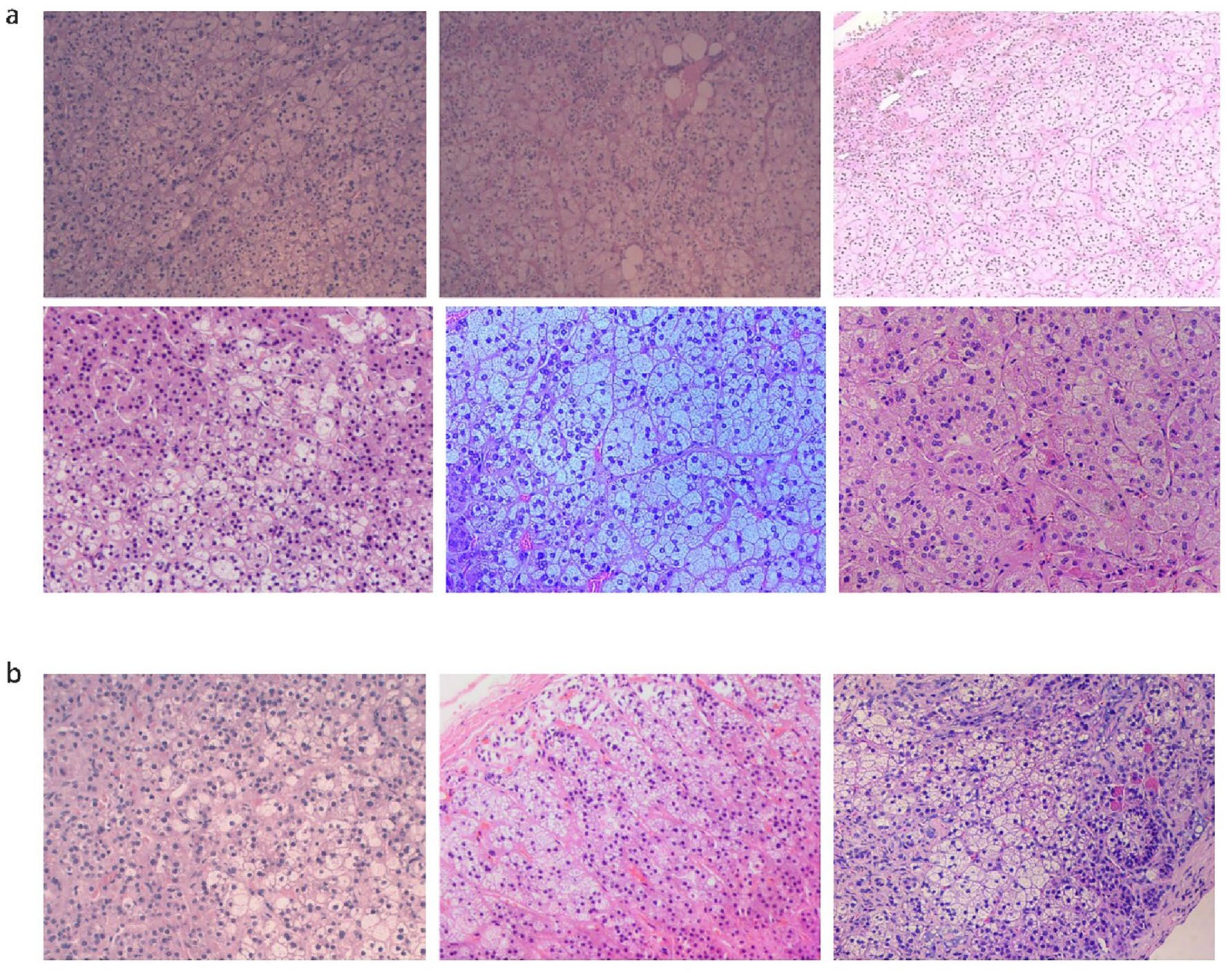
HE staining of the UPA patients. Scale bar, 50 μm. (**a**) The pathological result of part APA patients. (**b**) The pathological result of part UAH patients.

According to the pathological analysis of 18 patients, we found that there were two pathological types including adrenocortical adenoma and adrenocortical hyperplasia in our study. Therefore, the unilateral PA patients were further divided into adenoma and hyperplasia group according to the pathological type (Fig. 3), and by comparing the clinical information between adenoma and hyperplasia group (Table 4), we obtain valuable clinical indicators like fasting glucose, glycosylated hemoglobin, which were higher in hyperplasia than adenoma (P < 0.05), while plasma aldosterone supine was higher in adenoma group than hyperplasia. The remaining clinical indicators were not statistically significant (P > 0.05).

**Table 4:** Comparison of baseline data and cytokine levels between adenoma and hyperplasia groups.

	Adenoma (n = 13)	Hyperplasia (n = 5)	P
Clinical data			
ALD supine (pg/ml)	524.51 (285.31 ~ 783.62)	228.6 (122.16 ~ 326.25)	0.019
FBG (mmol/L)	4.52 (4.395 ~ 5.14)	5.06 (4.96 ~ 7.73)	0.035
HBALC (%)	5.3 (5.1 ~ 6)	6.1 (5.8 ~ 10.2)	0.046
Middle			
IL-1b (pg/ml) 4.7	(2.825 ~ 6.01)	7.6 (5.6 ~ 54.6)	0.026
IL-8 (pg/ml)	4.1 (2.365 ~ 6.435)	9.3 (7.3 ~ 147.6) .007	
MIP-1a (pg/ml)	15.2 (10.2 ~ 21.5)	27.28 (18.4 ~ 52.2)	0.035
MIP-1b (pg/ml)	36.3 (32.7 ~ 47.5)	28.5 (25.7 ~ 38.2)	0.035
Opposite			
IL-8 (pg/ml)	3.96 (2.235 ~ 4.64)	6.92 (6.02 ~ 63.765)	0.002
IL-1b (pg/ml)	4.25 (3.22 ~ 5.34)	6.92 (5.785 ~ 53.365)	0.010
TGFa (pg/ml)	3.01 (1.91 ~ 8.305)	11.11 (4.375 ~ 17.5)	0.046
Same			
IL-12p70 (pg/ml)	1.23 (0 ~ 1.805)	11.05 (1.115 ~ 29.86)	0.046
IL-17E (pg/ml)	110.5 (90.9 ~ 178.5)	267.6 (225.9 ~ 472.2)	0.026
IL-1b (pg/ml)	3.83 (3.02 ~ 5.01)	7.85 (7.155 ~ 62.775)	0.000
MIP-1a (pg/ml)	12.5 (10.5 ~ 16.0)	25.9 (18.4 ~ 57.9)	0.007
MIP-1b (pg/ml)	38.3 (36.5 ~ 42.7)	27.6 (24.6 ~ 32.4)	0.003

ALD supine: plasma aldosterone supine; FBG: fasting glucose; HBALC: glycosylated hemoglobin; Middle: Middle group; Opposite: Opposite group; Same: Same group.

P < 0.05 are considered significant.

### Comparison of adenoma and hyperplasia groups in 48 cytokines

The comparison of adenoma and hyperplasia groups in cytokines levels were conducted in the Middle, Same and Opposite groups, respectively (Table 4). In the Middle group, the levels of IL-1b, IL-8 and MIP-1a in the adenoma group were much lower than those in the hyperplasia group, and only MIP-1b was significantly higher than that in the hyperplasia group, while in the Opposite group, the levels of statistically significant indexes like IL-1b, IL-8 and TGF-a were also higher in the Hyperplasia group than in the Adenoma group. In the Same group, the levels of MIP-1b in the adenoma group were significantly higher than those in the hyperplasia group, while IL-12p70, IL-17E, IL-1b and MIP-1a were all lower than those in the hyperplasia group.

### The ability of IL-1b in plasma to differentiate adenoma from hyperplasia

**Figure 4:**
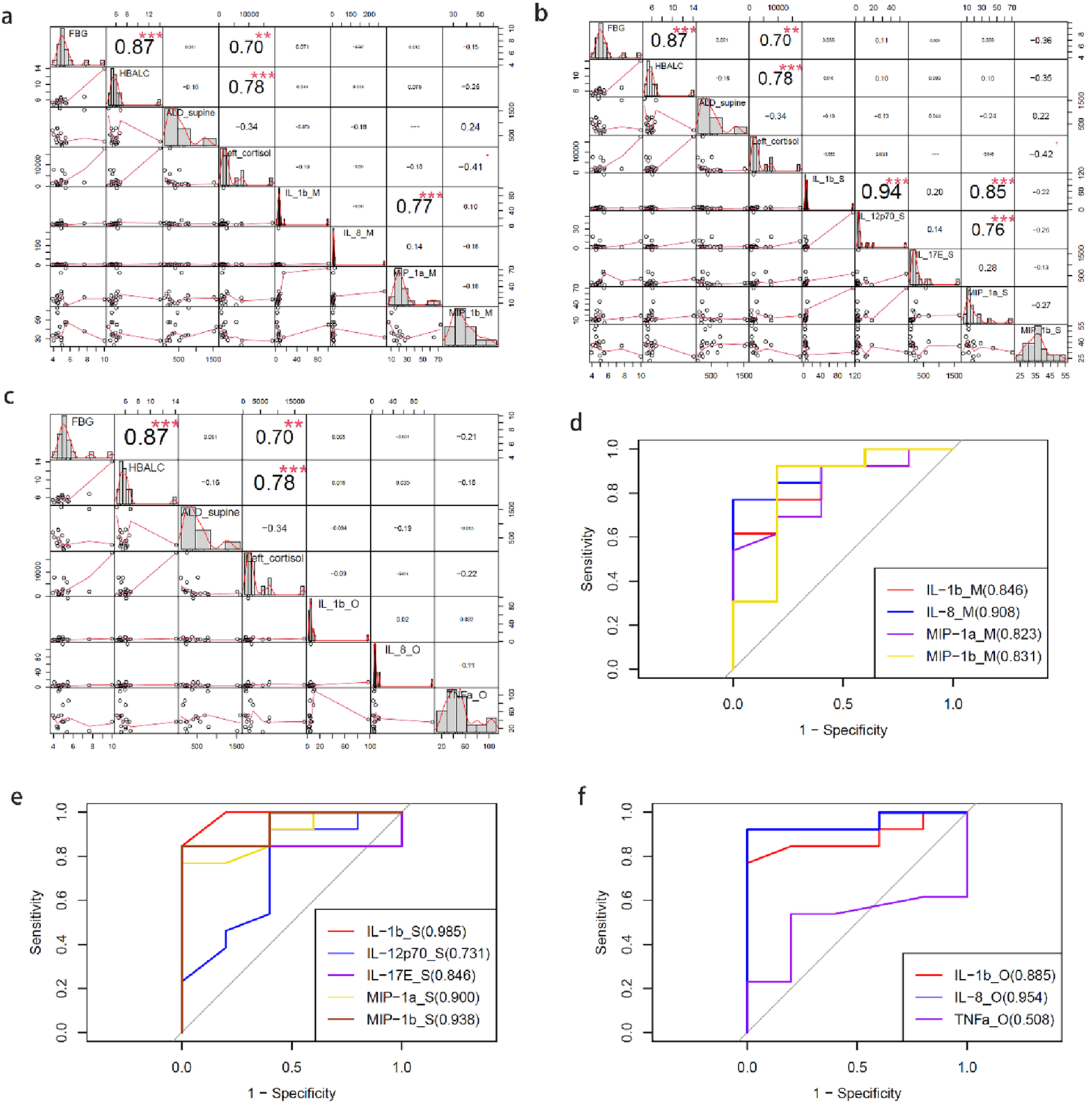
The correlation analysis between the clinical materials with the cytokines and the ROC curve of different histological type prediction. (**a**) The correlation analysis in the Middle group. (**b**) The correlation analysis in the Same group. (**c**) The correlation analysis in the Opposite group. (**d**) The ROC curve in the Middle group. (**e**) The ROC curve in the Same group. (**f**) The ROC curve in the Opposite group.

The correlation analysis of adenoma and hyperplasia groups between cytokines levels and the clinical data were conducted in the Middle, Same and Opposite groups, respectively (Fig. 4a,b,c). However, the result of correlational analyses had no statistical significance (P > 0.05). In order to distinguish the two pathological patterns, the cytokines from three different subgroups (Opposite, Same, and Middle groups) were selected to paint ROC curves, separately (Fig. 4d,e,f). According to ROC curves, IL-1b was suggested to be the most potential diagnostic biomarker to discriminate the two types, because the three subgroups all showed the values of AUC were all over 0.84 and plasma IL-1b can discriminate the hyperplasia from adenoma patients at over 61.5% of sensitivity, 100% of specificity.

### The correlational analyses in clinical indicators and some cytokines of adenoma and hyperplasia

The correlational analyses were conducted between the important cytokines and the clinical indicators (Fig. 4a,b,c), However, the correlational analyses between clinical indicators and cytokines were not reach statistical significance (p > 0.05).

### The immunohistochemical staining of IP-10, CXCL9, RANTES and IL-1 in different groups

**Figure 5:**
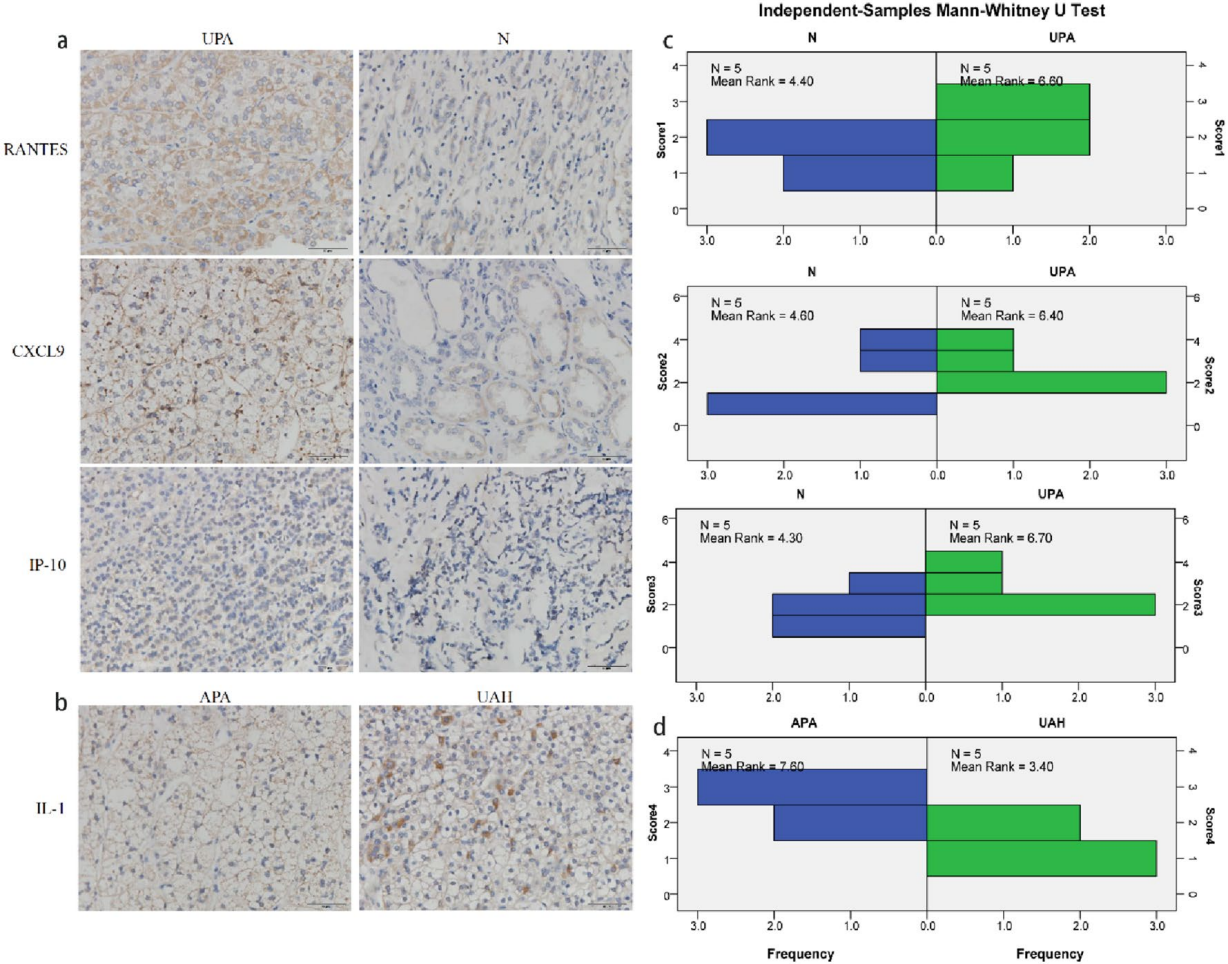
(**a**) The sections are shown at 40 × magnification. The expressions of RANTES, CXCL9, and IP-10 were visible in adrenal cells (brown cytoplasm). (**b**) The sections are shown at 40 × magnification. The expression of IL-1 was visible in adrenal cells (brown cytoplasm). (**c**) The result of Mann–Whitney test between UPA and control group. (**d**) The result of Mann–Whitney test between APA and UAH group.

The results of immunohistochemical staining were listed in Table 5. The comparison between UPA and normal adrenal in RANTES, CXCL9 and IP-10 tissue expressions became no significant difference (all p > 0.05), although the expression of RANTES, CXCL9 and IP-10 in the UPA all exhibited higher levels than normal ones. However, the tissues expression of IL-1 in UAH patients was significantly reduced than APA patients (p < 0.05), which was different from the plasma comparison (Fig. 5a–d).

**Table 5:** The result of immunohistochemical staining.

Sample	RANTES		CXCL9		IP-10		Sample	IL-1	
	Range (%)	Score	Range (%)	Score	Range(%)	Score		Range (%)	Score
UPA1	20	1	40	2	30	2	APA1	40	2
UPA2	30	2	50	2	70	3	APA2	70	3
UPA3	60	3	70	3	40	2	APA3	70	3
UPA4	35	2	35	2	30	2	APA4	30	2
UPA5	55	3	90	4	80	4	APA5	70	3
N1	50	2	5	1	5	1	UAH1	20	1
N2	35	2	80	4	5	1	UAH2	10	1
N3	10	1	5	1	50	2	UAH3	50	2
N4	10	1	10	1	30	2	UAH4	40	2
N5	40	2	60	3	70	3	UAH5	5	1

UPA: unilateral primary aldosteronism; N: normal adrenal gland; APA: aldosterone-producing adenoma; UAH: unilateral adrenal hyperplasia; Range: the distribution range of positive cells; Score: on a scale of 1 to 4, the distribution of positive cells ranging 0–25% rated 1, ranging 26–50% rated 2, ranging 51–75% rated 3, and ranging 76–100% rated 4.

## Discussion

To my knowledge, this is the first study to combine AVS technology with MILLIPLEX immune kit technology, and the first to obtain blood samples closest to the adrenal gland for a broad profile of adrenal dominant and non-dominant cytokines, including chemokines and growth factors, and the first time to detect so many cytokines in the UPA patients and healthy people. We acquired the expression of cytokines in adrenal vein in UPA patients and obtain some meaningful results. As a special intervention method, AVS can obtain the blood tissue closest to the lesion, which has greater clinical significance compared with peripheral blood sampling, and specially the inferior venous blood will more accurately reflect systemic inflammatory factors when compared with the traditional elbow vein or other veins in upper limbs, meantime the blood samples closest to the adrenal glands could intuitively reflect the local changes. Therefore, by the comparison of inferior venous and adrenal vein blood, we will acquire the influence of local adrenal aldosteronoma to the systemic.

The result showed SBP, DBP, BMI, HR and TG in the UPA group were significantly increased, which was consistent with previous reports that patients with UPA both APA and UAH have increased blood pressure and blood lipid levels due to elevated aldosterone levels promoting sodium and water retention and the probability of metabolic syndrome than that in the normal population^16^. In our study, IP-10, CXCL9 and RANTES were significantly higher in the patients compared with the normal ones. RANTES known to expressed and secreted by activated and normal T cell^23^, IP-10 attracting activated T, and CXCL9 involving in T cell migration^24,25^, were all showing the close relationship with T cells, and those cytokines were strongly associated with CD8 + T-cell infiltration in solid tumors^26–28^. Early researches have detected the abundant lymphocytic infiltration in adrenocortical tumor, and recent single cell sequencing studies have further detected the CD8 + T cells with highest proportion in normal adrenal glands^7,29,30^, therefore we speculated that these inflammatory factors might play a role in unilateral PA formation.

According to ROC curves, the combination of IP-10, CXCL9 and RANTES might be reliable indexes as a biomarker to discriminate UPA patients from the healthy ones. As we all know, aldosterone also exerts a strong inflammatory effect and as a powerful immune regulator, and it could act on the mineralocorticoid receptor of immune cells to increase some inflammation factors expression^31^, like TGF-β1, TNF-α, IL-6, MCP-1^32–35^, which related to the mechanisms of fibrosis. Although a few early reports have conducted the research on the inflammation factors in APA like TNF-α and IL-6, the results were no value in APA prediction^36,37^, which was attributed to the researches were just limited to a small subset of cytokine. Therefore, we have expanded the inflammation factors research covered 48 cytokines, and finally found that IP-10, CXCL9 and RANTES might be reliable indexes as a biomarker to discriminate UPA patients from the healthy ones. Moreover, our correlational analyses of all the cohorts also demonstrated that IP-10 and CXCL9 were positively correlated with BP and HR, which implicated it might be a marker to reflect the therapeutic effect in UPA patients. Moreover, EGF positively correlated with HDL could also use as therapeutic effect in UPA patients. Although the expression of IP-10, CXCL9 and RANTES in the adrenal tissues of UPA and normal adrenal showed no statistical difference, which might attribute to the less samples in our study. However, we found that the expression of RANTES, CXCL9 and IP-10 in the UPA all exhibited higher levels than normal ones, which implicated those factors might be meaningful if more samples were included in the study.

Adrenal veins received the blood from the adrenal gland, the serum of which could reflect adrenal local lesions. AVS results have demonstrated aldosterone level in the dominant side is significantly elevating than that in non-dominant side in UPA patients^38^. In order to further study the difference of the dominant and non-dominant sides in adrenal gland, we collected the plasma from bilateral adrenal veins and the blood from inferior vena cava served as control group. Contrary to expectations, although the adrenal tissue in dominant side seemed to have greater inflammatory response than non-dominant sides, the level of cytokines between the two sides didn’t show any statistically significant. In addition, in the study on adrenal veins and inferior vena cava, the cytokines like FLT-3L, G-CSF, IL-15, M-CSF, and MDC were both higher in the two sides than peripheral blood. Notably, the elevating factors in the adrenal veins were macrophage and monocyte associated factors, including granulocyte colony stimulating factor (G-CSF), macrophage colony stimulating factor (M-CSF), macrophage-derived chemokine (MDC), monocyte chemokine protein-1 (MCP-1)^39–41^. In addition, other elevating cytokines such as FMS-like Tyrosine kinase 3 Ligand (FLT3L), IL-15 have also been identified as pro-inflammatory cytokines, mainly involved in the regulation of T cells, according to previous studies^42,43^,and normal adrenal gland microenvironment also demonstrated the high portion of macrophage^7^, which implicating the important function conducted by both macrophage and CD8 + T cells.

Beyond expectation, two pathological groups of UPA showed the differences in levels of 7 cytokines with statistical significance and most of elevating cytokines were higher in the Hyperplasia group than Adenoma group, which implicated the different pathological could lead to different inflammation change and hyperplasia pathological type might possess more strong inflammation response than adenoma. Furthermore, the comparison of baseline data of patients in different pathological types showed higher levels in the hyperplasia patients, like the levels of ALD supine, which further implicated the severer manifestation in the hyperplasia pathological type. However, the results in immunohistochemistry were different from the plasma, the IL-1 was higher in UAH serum than APA, while was decreased in UAH tissues than APA. The serum reflects the whole adrenal lesion, but the sections reflect the local tissue. Previous researches have found the same adrenal tissues might exit different nodules, and different lesion might possess different pathological mechanisms^44,45^, which the contradictory results might be attributed to it. Moreover, other clinical indexes like BP, serum potassium did not show any significant difference, which might attribute to quite a small sample, and more samples were acquired to add to support our result. According to the assessment by ROC curves, IL-1b might be suggested to be the potential diagnostic biomarker to discriminate the two types and more clinical trails need to conduct to confirm the effect in pathological type perdition.

## Limitations

Because of the expensive MILLIPLEX immune kit, just small numbers of cases were involved in the study, the results of which might not be accurate enough. Moreover, there are six types in the unilateral forms of PA, but only two main types of UPA were incorporated in our study out of multiple causes. Therefore, more experiments were required to conduct to further confirmed our conclusion. Moreover, this study didn't include bilateral PA in the limitation section, which is the largest bias in this study.

## Conclusion

In summary, our study was the first to combine AVS technology with MILLIPLEX immune kit technology, and by conducting broad profile of adrenal veins cytokines, our study found some cytokines might be used as a sign to help APA diagnosis or pathological classification, meantime, more experiment and clinical data were required to support our result in the future.

## Data Availability

The datasets used and analyzed during the current study available from the corresponding author on reasonable request.
